# Testing the 2018 NIA-AA research framework in a retrospective large cohort of patients with cognitive impairment: from biological biomarkers to clinical syndromes

**DOI:** 10.1186/s13195-019-0543-7

**Published:** 2019-10-15

**Authors:** Tiziana Carandini, Andrea Arighi, Luca Sacchi, Giorgio G. Fumagalli, Anna M. Pietroboni, Laura Ghezzi, Annalisa Colombi, Marta Scarioni, Chiara Fenoglio, Milena A. De Riz, Giorgio Marotta, Elio Scarpini, Daniela Galimberti

**Affiliations:** 1 Fondazione IRCCS Ca’ Granda Ospedale Maggiore Policlinico, Via F. Sforza 35, 20122 Milan, Italy; 2 Dino Ferrari Center, University of Milan, Milan, Italy; 3 Department of Neurosciences, Psychology, Drug Research and Child Health (NEUROFARBA), University of Florence, Florence, Italy

**Keywords:** Alzheimer’s disease, Dementia, CSF, Clinical neurology, Biomarkers, PET

## Abstract

**Background:**

According to the 2018 NIA-AA research framework, Alzheimer’s disease (AD) is not defined by the clinical consequences of the disease, but by its underlying pathology, measured by biomarkers. Evidence of both amyloid-β (Aβ) and phosphorylated tau protein (p-tau) deposition—assessed interchangeably with amyloid-positron emission tomography (PET) and/or cerebrospinal fluid (CSF) analysis—is needed to diagnose AD in a living person. Our aim was to test the new NIA-AA research framework in a large cohort of cognitively impaired patients to evaluate correspondence between the clinical syndromes and the underlying pathologic process testified by biomarkers.

**Methods:**

We retrospectively analysed 628 subjects referred to our centre in suspicion of dementia, who underwent CSF analysis, together with neuropsychological assessment and neuroimaging, and were diagnosed with different neurodegenerative dementias according to current criteria, or as cognitively unimpaired. Subjects were classified considering CSF biomarkers, and the prevalence of normal, AD-continuum and non-AD profiles in each clinical syndrome was calculated. The positivity threshold of each CSF biomarker was first assessed by receiver operating characteristic analysis, using Aβ-positive/negative status as determined by amyloid-PET visual reads. The agreement between CSF and amyloid-PET data was also evaluated.

**Results:**

Among patients with a clinical diagnosis of AD, 94.1% were in the AD-continuum, whereas 5.5% were classified as non-AD and 0.4% were normal. The AD-continuum profile was found also in 26.2% of frontotemporal dementia, 48.6% of Lewy body dementia, 25% of atypical parkinsonism and 44.7% of vascular dementia. Biomarkers’ profile did not differ in amnestic and not amnestic mild cognitive impairment. CSF Aβ levels and amyloid-PET tracer binding negatively correlated, and the concordance between the two Aβ biomarkers was 89%.

**Conclusions:**

The examination of the 2018 NIA-AA research framework in our clinical setting revealed a good, but incomplete, correspondence between the clinical syndromes and the underlying pathologic process measured by CSF biomarkers. The AD-continuum profile resulted to be a sensitive, but non-specific biomarker with regard to the clinical AD diagnosis. CSF and PET Aβ biomarkers were found to be not perfectly interchangeable to quantify the Aβ burden, possibly because they measure different aspects of AD pathology.

## Introduction

The diagnosis of probable Alzheimer’s disease (AD) requires core clinical criteria [1]. Cerebrospinal fluid (CSF) and neuroimaging biomarkers enhance the specificity of the criteria in clinical and research settings [1]. In 2018, the National Institute of Age-Alzheimer’s Association shifted the definition of AD to a biological construct [2]. According to the new research framework (2018-NIA-AA-RF), AD is not defined by its clinical consequences, but by its underlying pathology as measured during lifetime by biomarkers [2]. The amyloid/tau/neurodegeneration (AT(N)) classification is used to divide biomarkers into those measuring β-amyloid (Aβ) deposition (A) [CSF Aβ levels or Aβ-positron emission tomography (PET)], pathologic phosphorylated tau (T) [CSF phospho-tau (p-tau) levels or tau-PET], and neurodegeneration (N) [18F-fluorodeoxyglucose-PET (FDG-PET), magnetic resonance imaging (MRI), or CSF total tau (t-tau) levels] [3]. Regardless of the presence of clinical symptoms, both Aβ and p-tau pathology are required for classification as AD, whereas Aβ deposition alone is an early sign, labelled AD pathologic change [2]. CSF and PET biomarkers are considered interchangeable in demonstrating AD pathology, assuming that they provide the same information [2]. Nevertheless, while Aβ-PET tracers bind to Aβ fibrils and give both quantitative and qualitative data about the topology of Aβ deposition, CSF biomarkers do not provide any topological information. Moreover, Aβ-PET can be easily interpreted as positive or negative by visual inspection, whereas general cut-offs for CSF biomarkers are lacking and the existing ones show high variability among centres [4–6]. Standardisation of measurements and protocols are ongoing [7].

The 2018-NIA-AA-RF retains also a staging for clinical severity, ranging from cognitively unimpaired (CU) to mild cognitive impairment (MCI) and dementia [2]. However, the criteria are currently supposed to be used in research setting only, and their validity needs to be examined before adopted into clinical practice [2].

The aim of this work was to test the 2018-NIA-AA-RF in the clinical scenario of a large cohort of cognitively impaired patients to evaluate possible correspondence between the clinical syndromes and the underlying pathologic process testified by biomarkers. Due to the availability of our centre, we used only CSF data to classify subjects accordingly to the 2018-NIA-AA-RF. CSF biomarker positivity thresholds for subject dichotomization were first assessed in a subgroup of subjects who underwent both CSF analysis and 18F-florbetapir-PET (amyloid-PET).

## Methods

### Design of the study

We tested the 2018-NIA-AA-RF by retrospectively analysing all subjects who underwent lumbar puncture (LP) for diagnostic purpose in the AD Unit of the Ospedale Maggiore Policlinico, University of Milan, Italy, from June 2011 to December 2017. Participants were referred to our centre in suspicion of dementia, and they all received—in addition to LP—a complete neurological examination, neuropsychological assessment and neuroimaging (brain MRI and/or FDG-PET). All the exams were performed within a 365-day interval from subjects’ first visit. After the diagnostic work-up, subjects were diagnosed by expert neurologists with MCI, or dementia, according to the specific criteria of each syndrome [1, 8–17]. A few individuals were instead CU. Regarding AD, from 2011 to 2014, clinical diagnoses were supported by CSF (Aβ_1-42_, p-tau and t-tau) and/or neuroimaging (brain MRI and/or FDG-PET) biomarkers, according to previous criteria [1, 9]. After the publication of the IWG-2 criteria in 2014 [10], CSF analysis or amyloid PET were used to support the diagnosis of probable AD in our clinical setting*.* Conversely, in line with their current criteria [11–17], all the other dementia syndromes were diagnosed combining clinical, neuropsychological and neuroimaging profiles, and CSF biomarkers were used for excluding AD.

According to 2018-NIA-AA-RF, CSF biomarkers were used to classify subjects into three binary categories: A+/−, T+/− and N+/−, respectively. Participants had three possible biomarker profiles and eight combinations: (1) normal [A−T− (N−)]; (2) AD-continuum ([A+T−(N−)], [A+T+(N−)], [A+T+(N+)] and [A+T−(N+)]); and (3) non-AD pathologic change (non-AD) ([A−T+(N−)], [A−T−(N+)] and [A−T+(N+)]) [2]. The positivity thresholds for subject dichotomization were first assessed in a subgroup of our cohort who underwent both LP and amyloid-PET within a 365-day interval in our unit for research purpose. In line with previous literature [4, 5, 10, 18, 19], we used Aβ-positive/negative status—as determined by amyloid-PET visual reads—to define our CSF positivity thresholds. We considered amyloid-PET the most suitable surrogate in vivo marker for determining the amyloid burden due to its high correlation with neuropathological results [4, 20–23].

### CSF analysis

CSF samples were collected by LP in the L3/L4 or L4/L5 interspace and centrifuged in 8000 rpm for 10 min. The supernatants were aliquoted in polypropylene tubes and stored at − 80 °C until use. CSF Aβ_1-42_, p-tau and t-tau were measured by using the commercially available sandwich enzyme-linked immunosorbent assay kits (Fujirebio, Ghent, Belgium). We also calculated the ratios of t-tau/Aβ_1-42_ and p-tau/Aβ_1-42_.

### Amyloid-PET imaging

Amyloid-PET scans were obtained with a Biograph Truepoint 64 PET/CT scanner (Siemens, Erlangen, Germany). All patients underwent PET scanning at rest after intravenous injection of 370 MBq. Amyloid-PET data were first qualitatively analysed by a trained physiologist using a binary method of interpretation for relating “positive” or “negative” scans to neuropathologically defined categories of Aβ plaque density. Structural MRI was also acquired, and FLAIR-weighted images and PET images were co-registered to individual volumetric T1-weighted images. After PET/MRI co-registration, ImcCalc of Statistical Parametric Mapping (SPM12, Wellcome Department of Cognitive Neurology, London, UK) was used to derive standardised uptake value (SUV) PET maps as SUV = AC/(radiotracer dose/BW). AC represents activity concentration in a given voxel [kBq/ml], radiotracer dose is the injected tracer dose corrected for residual activity in the syringe [MBq] and BW is the body weight [kg]. SUV maps were calculated in the grey matter (GM) of anterior cingulate gyrus, frontal lobe, parietal lobe, posterior cingulate gyrus, precuneus, temporal lobe, and of the average of these six regions (GM mean). The whole cerebellum was the reference region for the SUV relative ratio (SUVR). For the GM mean, we applied the validated threshold for amyloid-PET SUVR (1.11) [24, 25].

### Statistical analysis

All statistical analyses were conducted with Microsoft-Excel 2011, SPSS 21.0 (SPSS Inc., Chicago, IL, USA), or Graph-Pad-PRISM 6.0 (GraphPad Software, La Jolla, CA). Comparisons between amyloid-PET-positive (aP+) and amyloid-PET-negative (aP−) subjects were performed using non-parametric unpaired *t* tests (Mann-Whitney *U* test), except sex difference between aP+ and aP− that was tested by *Χ*^2^ test. Using receiver operating characteristic (ROC) analysis, we calculated the area under the curve (AUC) of the CSF analyte and ratio that best distinguished aP+ from aP− subjects. For each parameter, sensitivity was defined as the positivity rate in aP+ subjects, and specificity as the negativity rate in aP− subjects. The value of each CSF analyte or ratio with the highest Youden index (sensitivity + specificity − 1) was selected as the cut-off. The prevalence of normal, AD-continuum and non-AD in each clinical syndrome and in aP+ and aP− subjects was calculated and compared by *Χ*^2^ test. Correlations between amyloid-PET SUVR and CSF biomarkers were performed using Spearman coefficient, assuming a non-normal distribution of data. The concordance between amyloid-PET profile and CSF profile was defined as the sum of aP+ with pathologic CSF analyte or ratio levels and aP− with normal CSF analyte or ratio levels, divided by the entire cohort size. For all the analyses, we set the statistical threshold at *p* <  0.05.

### Determination of positivity thresholds for CSF biomarkers

Forty-four subjects underwent amyloid-PET and LP within a 365-day period for research purpose and were considered to identify positivity thresholds for CSF biomarkers. Amyloid-PET was visually assessed as positive in 37 patients (aP+) and negative in 7 (aP−). Accordingly, GM mean SUVR was > 1.11 and ≤ 1.11 in all aP+ and aP− participants, respectively. The AUC for the detection of amyloid-PET positivity was 0.86 (95% CI 0.71–1.00, *p* = 0.002) for Aβ_1-42_ and 0.80 (95% CI 0.58–1.03, *p* = 0.01) for p-tau/Aβ_1-42_. The following cut-offs that maximised the Youden index for predicting amyloid-PET positivity were selected: Aβ_1-42_ < 660 pg/ml (sensitivity 0.89, specificity 0.71) and p-tau/Aβ_1-42_ > 0.09 (sensitivity 0.81, specificity 0.71, Additional file 1). In line with the 2018-NIA-AA-RF [2], Aβ_1-42_ (cut-off < 660 pg/ml) resulted a better biomarker of β-amyloidosis (A)—as compared with p-tau/Aβ_1-42_, due to the higher accuracy, sensitivity and specificity in detection of amyloid-PET positivity. Conversely, ROC analyses showed that t-tau and p-tau (and t-tau/Aβ_1-42_) had an insufficient accuracy in distinguishing aP+ from aP− subjects (AUC 0.52, 0.61, 0.68, respectively; data not significant). Thus, we decided to not calculate cut-offs for these biomarkers, but to use standardly used cut-offs [26] due to the (1) lack of tau-PET, FDG-PET and/or quantitative MRI data and (2) inaccuracy we found in the detection of amyloid-PET positivity of these biomarkers. In conclusion, the following thresholds were applied for patient dichotomization: Aβ_1-42_ < 660 pg/ml (A), p-tau > 61 pg/ml (T) and t-tau > 500 pg/ml (N). The main characteristics of aP+ and aP− subjects are summarised in Table 1.

**Table 1:** Characteristics of subjects who underwent 18F-florbetapir-positron emission tomography (amyloid-PET) and lumbar puncture (LP) within a 365-day interval

	Amyloid-PET positive*	Amyloid-PET negative°	*p* value
*n*	37	7	
Age	71.4 ± 7.5	72.9 ± 3.6	0.76
M:F	13:24	3:4	0.69
Interval LP/amyloid-PET (days)	168.6 ± 119.5	246.4 ± 115.1	0.35
Aβ_1-42_ (pg/ml)	542.2 ± 119.2	842.3 ± 341.9	*0.001*
t-tau (pg/ml)	700.5 ± 493.3	681.3 ± 593.1	0.83
p-tau (pg/ml)	83.9 ± 38.5	67 ± 31.8	0.34
t-tau/Aβ_1-42_	1.30 ± 0.9	0.90 ± 1.0	0.13
p-tau/Aβ_1-42_	0.15 ± 0.07	0.09 ± 0.06	*0.01*
Amyloid-PET SUVR GM mean	1.47 ± 0.21	0.98 ± 0.10	*< 0.0001*
Amyloid-PET SUVR Ant Cing	1.49 ± 025	0.99 ± 0.16	*< 0.0001*
Amyloid-PET SUVR frontal	1.36 ± 0.25	0.89 ± 0.19	*< 0.0001*
Amyloid-PET SUVR parietal	1.28 ± 0.19	1.04 ± 0.15	*0.005*
Amyloid-PET SUVR post Cing	1.55 ± 0.22	0.97 ± 0.20	*< 0.0001*
Amyloid-PET SUVR precuneus	1.61 ± 0.27	1.02 ± 0.22	*< 0.0001*
Amyloid-PET SUVR temporal	1.50 ± 0.19	1.04 ± 0.15	*< 0.0001*

Data are expressed as mean ± SD, unless otherwise specified. *p* values by unpaired *t* test. *Among amyloid-PET-positive patients, 14 were diagnosed of Alzheimer’s disease, 2 cerebral amyloid angiopathy, 20 mild cognitive impairment (MCI) and 1 mixed dementia. °Four amyloid-PET-negative patients had a diagnosis of MCI, 1 frontotemporal dementia, 1 mixed dementia and 1 dysthymic dementia

All significant data (*p* value < 0.05) are presented in italic

*Abbreviations*: *M* males, *F* females, *Aβ*_*1-42*_ amyloid-β_1-42_, *t-tau* total tau, *p-tau* phosphorylated tau, *SUVR* standardised uptake value relative ratio, *GM* grey matter

## Results

### Application of the 2018-NIA-AA-RF into clinical practice

CSF data from 628 subjects were retrospectively analysed (Table 2). Among the 229 patients with a clinical diagnosis of AD [1, 8–10], 162 had AD typical core criteria, 36 logopenic primary progressive aphasia (PPA) [11], 23 posterior cortical atrophy (PCA) [27] and 8 frontal variant AD (fv-AD) [10]. Among the 107 subjects diagnosed as frontotemporal dementia (FTD), 82 had behavioural variant (bv)-FTD [12], 11 non-fluent PPA [11] and 14 semantic PPA [11]. We included also 37 Lewy body dementia (LBD) [13], 12 atypical parkinsonism [progressive supranuclear palsy (PSP) [16] or corticobasal syndrome (CBS) [14]], 5 idiopathic Parkinson’s diseases [15], 67 vascular/mixed dementia (VaD/mixed) [17] and 30 other dementia (see Table 2 for details). MCI (*n* = 132) were divided in amnestic (*n* = 99; aMCI) and not amnestic (*n* = 33; naMCI) [28, 29]. Nine individuals were diagnosed as CU and remained CU after at least 24-month follow-up.

**Table 2:** Demographic data and AT(N) cerebrospinal fluid biomarker profiles of all included patients, divided according to their clinical syndrome

				Normal^ (*n* = 132)	AD-continuum^ (*n* = 389)				Non-AD^ (*n* = 107)		
	*n*	Age*	M:F	A−T−(N−)	A+T−(N−) AD pathologic change	A+T+(N−) AD	A+T+(N+) AD	A+T−(N+) AD and non-AD pathologic change	A−T+(N−)	A−T−(N+)	A−T+(N+)
AD	229	72 ± 8	96:133	1 (0.4)	56 (24.5)	34 (14.9)	118 (51.6)	7 (3.1)	5 (2.1)	1 (0.4)	7 (3.0)
FTD	107	73 ± 7	61:46	39 (36.5)	11 (10.4)	7 (6.5)	9 (8.4)	1 (0.9)	15 (14.0)	4 (3.7)	21 (19.6)
LBD	37	76 ± 5	20:17	13 (35.1)	9 (24.3)	4 (10.8)	3 (8.1)	2 (5.4)	2 (5.4)	1 (2.7)	3 (8.1)
PSP/CBS	12	69 ± 7	6:6	9 (75.0)	1 (8.3)	–	2 (16.7)	–	–	–	–
PD	5	62 ± 18	2:3	4 (80.0)	1 (20%)	–	–	–	–	–	–
VaD/mixed	67	76 ± 6	37:30	17 (25.4)	16 (23.9)	6 (8.9)	8 (11.9)	–	10 (14.9)	–	10 (14.9)
Others°	30	–	–	15 (50.0)	7 (23.3)	1 (3.3)	2 (6.7)	–	1 (3.3)	1 (3.3)	3 (10.0)
MCI	132	73 ± 7	62:70	27 (20.4)	35 (26.5)	18 (13.6)	25 (18.9)	4 (3.0)	11 (8.3)	–	12 (9.2)
CU	9	69 ± 6	8:1	7 (77.8)	2 (22.2)	–	–	–	–	–	–
Total	628			132 (21.0)	138 (22.0)	70 (11.1)	167 (26.6)	14 (2.2)	44 (7.0)	7 (1.1)	56 (9.0)

*Data are expressed as mean ± standard deviation. ^Data are expressed as number/total (percentage). °Among the 30 patients classified as “others” dementia, 4 had a diagnosis of sporadic cerebral amyloid angiopathy, 12 dysthymic dementia, 3 prion diseases, 1 Huntington disease, 1 Nasu-Hakola disease, 7 normal pressure hydrocephalus and 2 metabolic dementia

*Abbreviations*: *AD* Alzheimer’s disease, *M* males, *F* females, *FTD* frontotemporal dementia, *LBD* Lewy body dementia, *PSP* progressive supranuclear palsy, *CBS* corticobasal syndrome, *PD* idiopathic Parkinson’s disease, *VaD* vascular dementia, *MCI* mild cognitive impairment, *CU* cognitively unimpaired

According to their CSF data, participants were divided in normal (*n* = 132), AD-continuum (*n* = 389) and non-AD (*n* = 107) [2, 3]. The demographic data and a detailed list of biomarker profiles are given in Table 2.

### Correspondence between biological biomarkers and clinical syndromes

**Fig. 1:**
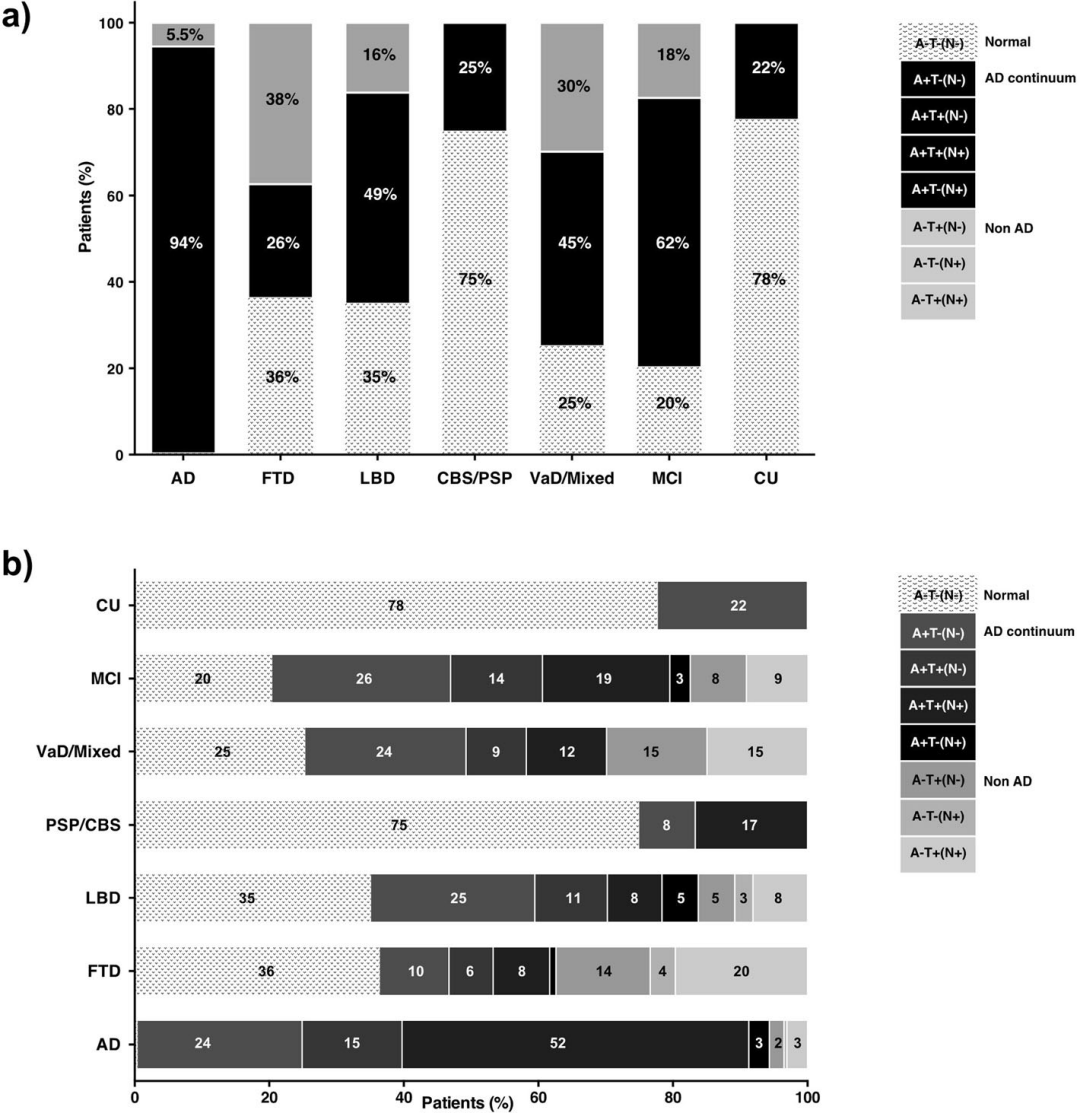
**a** Percentages of the three AT(N) biomarker profiles (AD-continuum, non-AD and normal) in each clinical syndrome. **b** Percentages of all the eight AT(N) biomarker profiles in each clinical syndrome. Percentages < 1% are not shown. AD Alzheimer’s disease (*n* = 229), FTD frontotemporal dementia (*n* = 107), LBD Lewy body dementia (37), PSP progressive supranuclear palsy (*n* = 3), CBS corticobasal syndrome (*n* = 9), VaD/mixed vascular/mixed dementia (*n* = 67), MCI mild cognitive impairment (*n* = 132), CU cognitively unimpaired (*n* = 9)

**Fig. 2:**
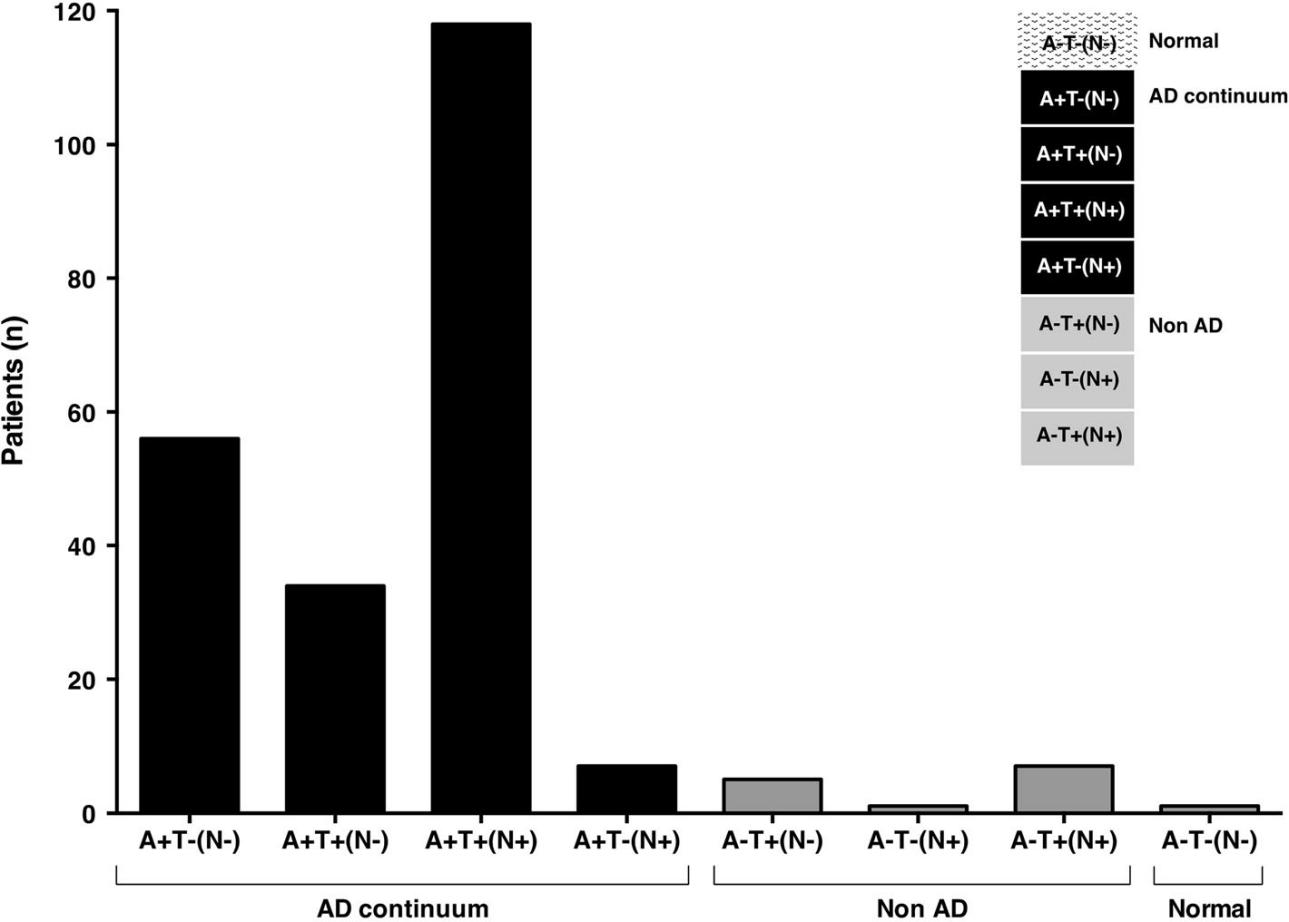
Number of Alzheimer’s disease-diagnosed patients (*n* tot = 229) for all the eight AT(N) biomarker profiles

The percentages of the three main biomarker profiles (normal, AD-continuum and non-AD) in each clinical syndrome are summarised in Fig. 1a, whereas Fig. 1b and Table 2 provide a detailed representation of the percentages of all the eight biomarker profiles in the different clinical syndromes considered. Among patients diagnosed with AD, 94.1% were in the AD-continuum according to 2018-NIA-AA-RF [2], whereas 5.5% were classified as non-AD and 0.4% were normal. Particularly, the AD profile was the most common in AD-diagnosed patients (A+T+(N−) = 14.9% and A+T+(N+) = 51.6%), followed by the AD pathologic change profile (A+T−(N−) = 24.5%). Only 3.1% displayed an AD and non-AD pathologic change profile. As concern AD-diagnosed patients with a non-AD profile, 2.1% was A−T+(N−), 0.4% A−T−(N+) and 3% A−T+(N+) (Fig. 2). The AD-continuum profile was significantly higher in patients with clinically diagnosed AD as compared with all the other diagnostic groups (*Χ*^2^ = 175.1; df = 4; *p* <  0.0001). When considering all the eight biomarker subgroups, the same trend was shown, although the significance threshold was not reached (*p* > 0.05).

Similar percentages were found when considering only typical AD [AD-continuum = 95%; non-AD = 5%] and logopenic PPA [AD-continuum = 92%; non-AD = 5.5%; normal = 2.5%]. PCA resulted AD-continuum and non-AD in 87% and 13% of cases, respectively. All fv-AD were AD-continuum.

37.3% of FTD-diagnosed patients were non-AD (A−T+(N−): 14%, A−T−(N+): 3.7%, A−T+(N+): 19.6%), and 36.5% were normal (36.5%). Nevertheless, 26.2% of FTD had an AD-continuum profile (A+T−(N−) = 10.4%, A+T+(N−) = 6.5%, A+T+(N+) = 8.4%, A+T−(N+) = 0.9%).

Among LBD patients, 48.6% was AD-continuum (A+T−(N−) = 24.3%, A+T+(N−) = 10.8%, A+T+(N+) = 8.1%, A+T−(N+) = 5.4%), 35.1% normal and 16.2% non-AD (A−T+(N−) = 5.4% A−T−(N+) = 2.7%, A−T+(N+) = 8.1%); 75% of CBS/PSP patients was normal, and 25% AD-continuum (A+T−(N−) = 8.3%, A+T+(N+) = 16.7%). Between VaD/mixed patients, 44.7% was AD-continuum (A+T−(N−) = 23.9%, A+T+(N−) = 8.9%, A+T+(N+) = 11.9%), 29.8% non-AD (A−T+(N−) = 14.9%, A−T+(N+) = 14.9%) and 25.4% normal. Seven out of the 9 CU had a normal profile (77.8%), but 2 CU subjects had AD pathologic change profile (A+T−(N− 22.2%)) [2], without clinical symptoms after 48-month follow-up.

**Fig. 3:**
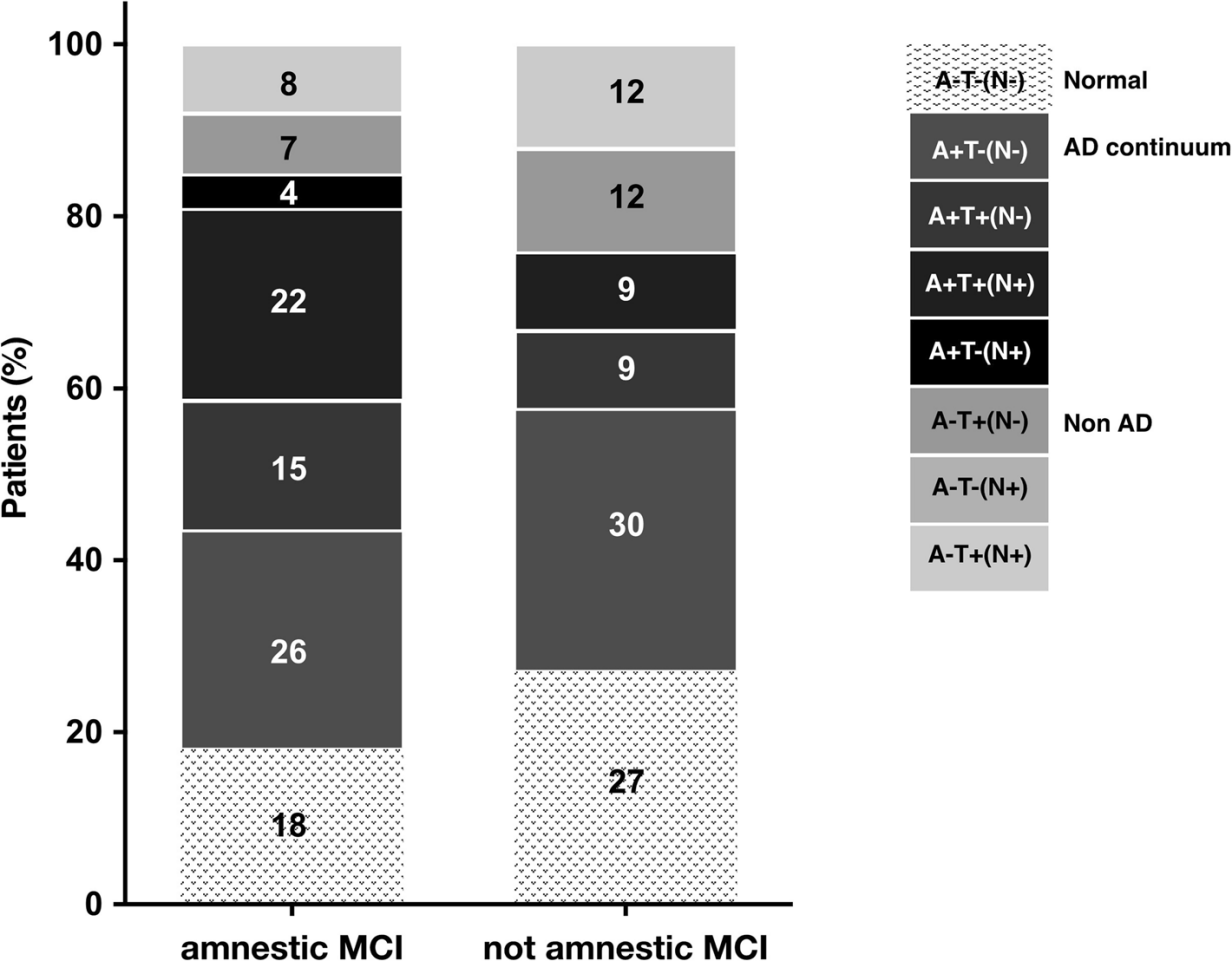
Percentages of all the eight AT(N) biomarker profiles in amnestic (*n* = 99) and not amnestic (*n* = 33) mild cognitive impairment (MCI) (*n* tot = 132)

MCI-diagnosed patients displayed more often an AD-continuum profile (62%; A+T−(N−) = 26.5%, A+T+(N−) = 13.6%, A+T+(N+) = 18.9%, A+T−(N+) = 3%), but 20.4% was normal, and 17.5% non-AD (A−T+(N−) = 8.3%, A−T+(N+) = 9.2%). When dividing MCI patients in aMCI and naMCI (Fig. 3), the AD-continuum was more common in aMCI (67% versus 49%; *X*^2^ not significant; *p* > 0.05). According to 2018-NIA-AA-RF [2], 37% of aMCI and 18% of naMCI were classified as AD with MCI (A+T+(N−)/A+T+(N+)), whereas 25% of aMCI and 30% of naMCI displayed the AD pathologic change profile (A+T−(N−)). The prevalence of non-AD was 15% in aMCI and 24% in naMCI, whereas 18% of aMCI and 27% of naMCI had the normal profile (Fig. 3).

### Correlations between amyloid biomarkers

CSF Aβ_1-42_ levels and p-tau/Aβ_1-42_ ratio were significantly different in aP+ and aP− groups (Table 1). CSF Aβ_1-42_ levels—but not p-tau/Aβ_1-42_ ratio—negatively correlated with amyloid-PET SUVR in GM mean (*r* = − 0.31, *p* = 0.039) and precuneus (*r* = − 0.30, *p* = 0.04). No correlation between CSF and PET data and no significant differences in aP+ and aP− patients were found for t-tau, p-tau and t-tau/Aβ_1-42_ (*p* > 0.05).

When CSF biomarkers were dichotomized according to the previously determined cut-offs, Aβ_1-42_ displayed the higher concordance between amyloid-PET and CSF data (89%), compared to p-tau/Aβ_1-42_ ratio (73%). Among aP+ patients, 89% (*n* = 33/37) was classified as AD-continuum by their CSF Aβ_1-42_ levels, 8% (*n* = 3/37) as non-AD and 3% (*n* = 1/37) as normal. Conversely, 43% (*n* = 3/7) and 28.5% (*n* = 2/7) of aP− participants had non-AD and normal CSF profiles, respectively, but 28.5% (*n* = 2/27) was AD-continuum (Additional file 2).

#### Correlation between Aβ_1-42_ and p-tau/Aβ_1-42_

According to our previously determined cut-off, 382 out of 628 subjects had a pathologic p-tau/Aβ_1-42_ ratio (> 0.09), as compared with the 389 participants with pathologic CSF Aβ_1-42_ levels. When considering p-tau/Aβ_1-42_ ratio—instead of Aβ_1-42_ levels—to classify patients according to the 2018-NIA-AA-RF [2], 90.4% of clinically diagnosed AD patients resulted AD-continuum (*n* = 207/229), 7.9% normal (*n* = 18/229) and 1.7% non-AD (*n* = 4/229). As concerns the other forms of dementia, when using p-tau/Aβ_1-42_ ratio, the percentage of AD-continuum was 40.1% in FTD patients (*n* = 43/107), 37.8% in LBD (*n* = 14/37), 16.7% in CBS/PSP (*n* = 2/12), 0% in PD (*n* = 0/5), 43.3% in VaD/mixed (*n* = 29/67), 61.4% in MCI (*n* = 81/132) and 0% in CU (*n* = 0/9; Additional file 3).

## Discussion

The 2018-NIA-AA-RF promotes a biological definition of AD based on its underlying pathologic process as measured by biomarkers. Evidence of both Aβ and pathologic p-tau deposition—assessed interchangeably with PET and/or CSF analysis—is needed to define AD in a living person [2, 4].

We first used amyloid-PET to calculate the CSF biomarker positivity thresholds for patient dichotomization as required by the AT(N) classification [3]. ROC analyses revealed that the optimal Aβ_1-42_ cut-off to predict PET positivity was < 660 pg/ml (sensitivity 89%, specificity 71%). This is in line with Palmqvist and colleagues who described an optimal cut-off for CSF Aβ_1-42_ of < 647 pg/ml (sensitivity 95%, specificity 90%) [18]. Aβ_1-42_ presented the best AUC, the major concordance with PET data, and was the only biomarker that correlated with PET SUVR. As already described, we found that the accuracy of CSF Aβ_1-42_ to predict cortical Aβ deposition status did not increase if the ratio p-tau/Aβ_1-42_ was used [5, 18, 30]. Given all these reasons, low CSF Aβ_1-42_ levels resulted the best biomarker of β-amyloidosis. Conversely, p-tau, t-tau and t-tau/Aβ_1-42_ showed a bad diagnostic value in our ROC analyses, which is in accordance with some previous studies [18, 31, 32], but not with others [19, 33]. A possible explanation for such discrepancies may derive from differences in staging or severity between cohorts studied.

When we applied the 2018-NIA-AA-RF to our cohort of patients, we found a good—but incomplete—correspondence between the diagnoses of AD made according to the clinical follow-up and those made with CSF biomarker profiles. Among all AD patients, 94.1% displayed an AD-continuum profile, but 5.5% were classified as non-AD, and 0.4% as normal. Similar percentages were found when considering both typical AD and logopenic PPA. PCA had AD-continuum and non-AD CSF profiles in 87% and 13% respectively, in line with the knowledge that multiple pathologies can underlie PCA [27]. One reason for this partial correspondence could be that from 2011 to 2014, AD clinical diagnoses in our centre were supported by both CSF and neuroimaging biomarkers, according to previous criteria [1, 9]. Thus, some patients with neuroimaging findings highly suggestive of AD (i.e. decreased FDG-PET in temporo-parietal cortex and/or atrophy on MRI in medial, basal and lateral temporal lobe, and medial parietal cortex) have been diagnosed with probable AD, even with a normal CSF profile. After the publication of the IWG-2 criteria in 2014 [10], CSF analysis or amyloid PET were used to support the diagnosis of probable AD in our clinical setting.

Our analysis revealed a low specificity of the AD-continuum profile, since it was found in a significant number of other forms of dementia, such as 25% of CBS/PSP, 44.7% of VaD/mixed, 48.6% of LBD and 26.2% of FTD. Among the AD-continuum, the most common CSF biomarker profile in all these syndromes was the AD pathologic change (A+T−(N−)). The lack in specificity of CSF Aβ_1-42_ levels is in accordance with previous findings [34], suggesting that brain β-amyloidosis may be a common comorbidity in other neurodegenerative syndromes [34]. Aware that only neuropathological studies could help in understanding these findings, our analysis suggests that the AT(N)) classification has a limited utility for differential diagnosis in the clinic setting. Notably, as compared to CSF Aβ_1-42_ levels, p-tau/Aβ_1-42_ ratio revealed a lower sensitivity with regard to the clinical AD diagnosis, but a slight higher specificity in differentiating the other forms of dementia, except FTD. This is in line with previous studies, showing that p-tau/Aβ_1-42_ ratio has a higher accuracy in AD differential diagnosis, as compared to Aβ_1-42_ levels [6, 35]. However, differently from what was reported by De Souza et al., p-tau/Aβ_1-42_ ratio did not appear a useful tool to distinguish AD from FTD [36].

As regards MCI, aMCI represents an early stage of AD, especially when associated with low CSF Aβ levels [37]. In our analysis, even though the AD-continuum profile was more common in aMCI compared to naMCI, data did not reach statistical significance and CSF profiles did not allow a substantial distinction between the two subgroups. Notably, AD pathologic change (A+/T−(N−))—that reflects Aβ deposition alone, thus representing an early sign of brain amyloidosis—did not appear the most represented profile in the aMCI group. Follow-up is needed to confirm the evolution of these patients, according to their baseline biomarker profile.

Recently, Jack et al. estimated in a large cohort of subjects the prevalence of three biomarker-based definitions of the AD-continuum from the NIA-AA-RF and compared it with the prevalence of three clinically defined diagnostic entities commonly associated with AD (MCI, dementia, and clinically defined probable AD). They found that biologically defined AD is more prevalent than clinically defined probable AD, possibly due to the asymptomatic individuals with biological AD [38]. Authors concluded that the two definitions create potential confusion around the term AD [38].

The last consideration regards the correspondence between CSF and PET Aβ biomarkers. As previously described [4, 19, 25, 31, 32, 39, 40], we confirmed a slight inverse correlation between CSF Aβ_1-42_ levels, and the binding of amyloid-PET tracer in total GM and precuneus. As speculated by Palmiqvist et al., this could indicate that CSF Aβ_1-42_ reflects the total aggregation status of Aβ_1-42_ in the whole brain [39]. When we applied the CSF Aβ_1-42_ cut-off based on amyloid-PET positivity, concordance was present in 89% of the subjects. Many different studies had previously investigated the agreement between PET and CSF data, reporting rates ranging from 72 to 92.4% [4, 5, 18, 25, 30, 33, 41]. The variable concordance between the two biomarkers has different explanations. Illàn-Gala et al. recently described a variable correlation between CSF Aβ_1-42_ levels and amyloid-PET data that was good in CU and MCI, but negligible in dementia [42]. Toledo and colleagues demonstrated a non-linear correlation between CSF and PET Aβ biomarkers [42, 43]. Recent studies have demonstrated that aP−/CSF+ subjects have increased rates of Aβ accumulation, and are likely to become aP+ [39, 44], suggesting that the two biomarkers measure AD pathology at different stages [4, 42]. CSF reflects soluble forms of Aβ that precede fibrillary deposition, and possibly becomes abnormal prior to PET [4, 39, 45]. In line with this observation, our two aP−/CSF+ patients had a diagnosis of aMCI and developed a clinically defined AD at follow-up. aP+/CSF− individuals have also been reported among both CU and CI [5, 7, 25, 46]. However, these subjects have usually elevated CSF t-tau and p-tau levels, and Aβ_1-42_ close to the cut-off, and are often diagnosed with MCI or AD. Different methodologies may also highly influence the proportions of CSF+ and/or aP+ subjects. Among the three aP+/CSF− participants we had in our study, two had elevated CSF t-tau and p-tau levels and one had nearly pathologic CFS Aβ_1-42_ levels (665 pg/ml). All of them had a clinical diagnosis of AD or mixed/VaD dementia. Lastly, the application of the 2018-NIA-AA-RF to those patients who performed both PET and CSF analysis confirmed a larger discordance in aP− participants, suggesting that the chosen CSF and neuroimaging markers of Aβ deposition are not perfectly interchangeable.

This work has several limitations. First, a gold standard for the assessment of cortical Aβ burden is lacking. In line with previous literature, we chose amyloid-PET as the best surrogate in vivo marker for determining the Aβ load because of its easy interpretation by visual inspection and its high correlation with neuropathological studies [5, 18, 20–23]. Even though ROC analyses and group comparisons are statistically significant, we are aware that there were relatively few aP− subjects, in comparison to aP+. The subgroup of participants with both CSF and PET data was small, and the average time interval between LP and amyloid-PET was of approximately 6 months. All these aspects might have influenced our findings. Moreover, the CSF positivity thresholds were defined based on maximising the discrimination between aP+ and aP− subjects, without an independent test set. Thus, the correspondence we described between CSF and PET data likely represents an upper limit. It should also be noted that the incorporation of biomarkers into some AD diagnosis may have biased the concordance between biomarker- and clinically based diagnoses. Due to lack of data, patients were classified considering CSF data only and Aβ deposition was measured without considering Aβ_42/40_ ratio, which is recently considered as having an improved diagnostic performance compared to Aβ_42_ alone [19]. Illàn-Gala et al. evaluated the consistency of the AT(N) classification with different biomarker combinations, finding important divergences and concluding that it does not achieve the required consistency to be used in clinical settings [42].

## Conclusions

The application of the new criteria to a large cohort of patients revealed a good, but incomplete, correspondence between the clinical syndromes and the underlying pathologic process as measured by CSF biomarkers. The AD-continuum profile resulted to be a sensitive, but non-specific biomarker with regard to the clinical AD diagnosis. The incomplete agreement we found between CSF and PET Aβ biomarkers suggests that they are not perfectly interchangeable to quantify the Aβ burden, possibly because they measure different features of AD pathology.

## Supplementary information

**Additional file 1.** Receiver operator characteristic (ROC) curves for Aβ_1-42_ levels, and p-tau/Aβ_1-42_ ratio compared to amyloid-PET tracer binding. Individuals were dichotomized into amyloid-PET positive and amyloid-PET negative as determined by PET visual reads. For each CSF biomarker, the table indicates the cut-off value and associated sensitivity (SE), specificity (SP), and area under the ROC curve (AUC) for the measure compared to amyloid-PET status. 95% confidence intervals are included in the parentheses.

**Additional file 2. **a) Column plot representing the 3 cerebrospinal fluid (CSF) biomarkers profiles in amyloid-PET positive (*n* = 37) and amyloid-PET negative (*n* = 7) subjects (n tot = 44). b) Column plot representing the percentage of amyloid-PET positive and amyloid-PET negative subjects in AD-continuum (*n* = 35), non-AD (*n* = 6), and Normal (n = 3) profiles, as assessed by CSF analyses.

**Additional file 3.** Comparison between the different percentages of subjects with an AD-continuum profile identified by using either cerebrospinal Aβ_1-42_ levels (black) or p-tau/Aβ_1-42_ ratio (white). [AD: Alzheimer’s disease; FTD: frontotemporal dementia; LBD: Lewy bodies dementia; PSP: progressive supranuclear palsy; CBS: corticobasal syndrome; PD: Parkinson’s disease; VaD/Mixed: vascular/mixed dementia; Other: other dementia syndromes; MCI: mild cognitive impairment; CU: cognitively unimpaired].

## Data Availability

The datasets used in this study are available from the corresponding author upon reasonable request.

## Abbreviations

2018-NIA-AA-RF: 2018 National Institute of Age-Alzheimer’s Association research framework
AD: Alzheimer’s disease
aMCI: Amnestic mild cognitive impairment
Amyloid-PET: 18F-florbetapir-positron emission tomography
aP−: Amyloid-PET negative
aP+: Amyloid-PET positive
AT(N): Amyloid/tau/(neurodegeneration)
AUC: Area under the curve
Aβ: β-amyloid
bv-FTD: Behavioural variant frontotemporal dementia
CBS: Corticobasal syndrome
CSF: Cerebrospinal fluid
CU: Cognitively unimpaired
FDG-PET: 18F-fluorodeoxyglucose-positron emission tomography
FTD: Frontotemporal dementia
fv-AD: Frontal variant AD
GM: Grey matter
LBD: Lewy body dementia
LP: Lumbar puncture
MCI: Mild cognitive impairment
MRI: Magnetic resonance imaging
naMCI: Not amnestic mild cognitive impairment
Non-AD: Non-AD pathologic change
PCA: Posterior cortical atrophy
PET: Positron emission tomography
PPA: Primary progressive aphasia
PSP: Progressive supranuclear palsy
p-tau: Phosphorylated tau protein
ROC: Receiver operating characteristic
SUV: Standardised uptake value
SUVR: Standardised uptake value relative ratio
t-tau: Total tau protein
VaD/mixed: Vascular/mixed dementia
