# Supplementary figures and images for: Testing the 2018 NIA-AA research framework in a retrospective large cohort of patients with cognitive impairment: from biological biomarkers to clinical syndromes
Source: Alzheimers Res Ther. 2019 Oct 15;11:84. doi: 10.1186/s13195-019-0543-7 (PMC6794758; doi:10.1186/s13195-019-0543-7)

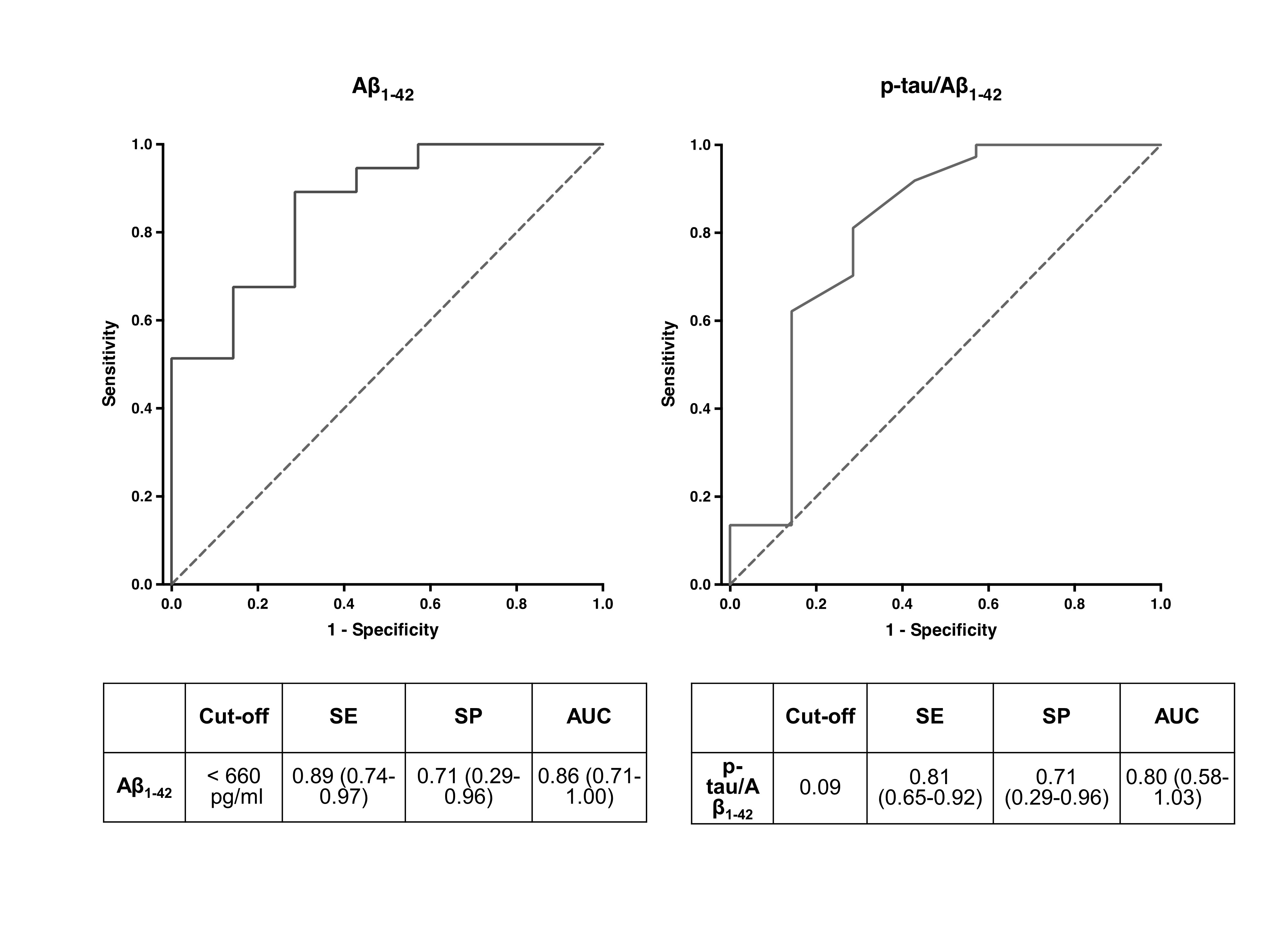

Supplement: Supplementary file 1 — Additional file 1. Receiver operator characteristic (ROC) curves for Aβ1-42 levels, and p-tau/Aβ1-42 ratio compared to amyloid-PET tracer binding. Individuals were dichotomized into amyloid-PET positive and amyloid-PET negative as determined by PET visual reads. For each CSF biomarker, the table indicates the cut-off value and associated sensitivity (SE), specificity (SP), and area under the ROC curve (AUC) for the measure compared to amyloid-PET status. 95% confidence intervals are included in the parentheses. [file 13195_2019_543_MOESM1_ESM.jpg]

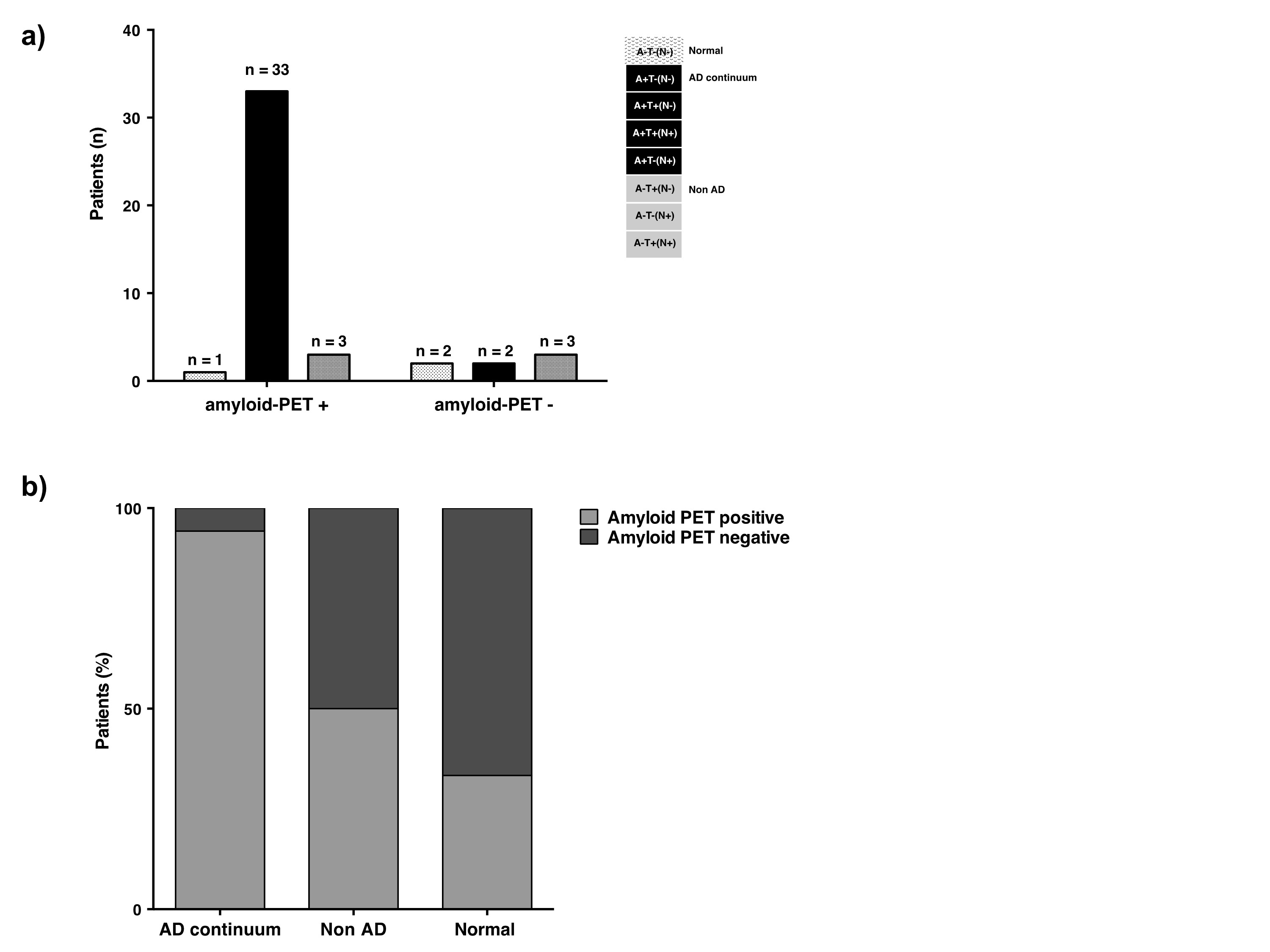

Supplement: Supplementary file 2 — Additional file 2. a) Column plot representing the 3 cerebrospinal fluid (CSF) biomarkers profiles in amyloid-PET positive (n = 37) and amyloid-PET negative (n = 7) subjects (n tot = 44). b) Column plot representing the percentage of amyloid-PET positive and amyloid-PET negative subjects in AD-continuum (n = 35), non-AD (n = 6), and Normal (n = 3) profiles, as assessed by CSF analyses. [file 13195_2019_543_MOESM2_ESM.jpg]

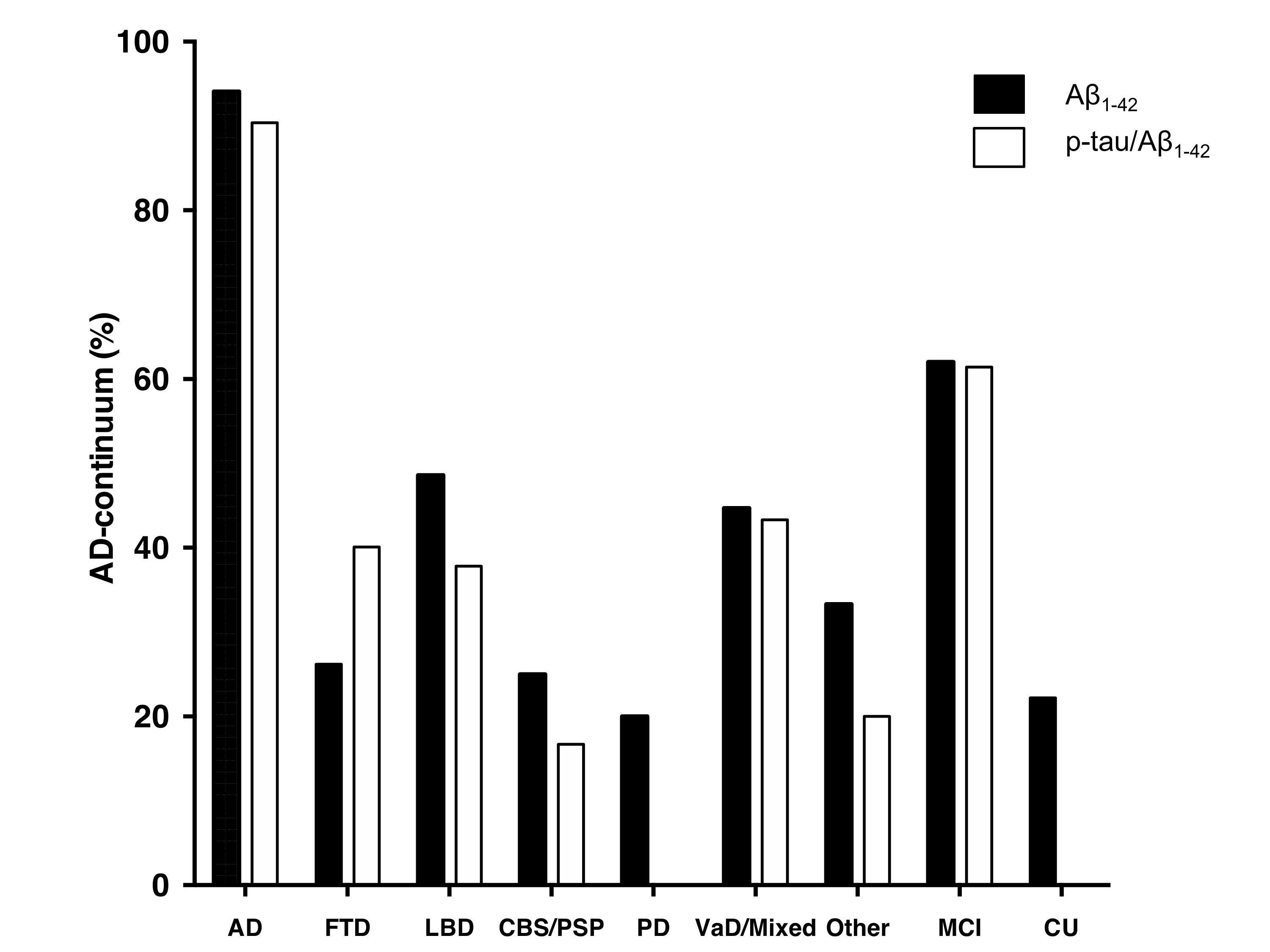

Supplement: Supplementary file 3 — Additional file 3. Comparison between the different percentages of subjects with an AD-continuum profile identified by using either cerebrospinal Aβ1-42 levels (black) or p-tau/Aβ1-42 ratio (white). [AD: Alzheimer’s disease; FTD: frontotemporal dementia; LBD: Lewy bodies dementia; PSP: progressive supranuclear palsy; CBS: corticobasal syndrome; PD: Parkinson’s disease; VaD/Mixed: vascular/mixed dementia; Other: other dementia syndromes; MCI: mild cognitive impairment; CU: cognitively unimpaired]. [file 13195_2019_543_MOESM3_ESM.jpg]
